# CD147 as a key mediator of the spleen inflammatory response in mice after focal cerebral ischemia

**DOI:** 10.1186/s12974-019-1609-y

**Published:** 2019-10-30

**Authors:** Rong Jin, Wei Zhong, Shan Liu, Guohong Li

**Affiliations:** 1 Department of Neurosurgery, Penn State Hershey Medical Center, 500 University Drive, Hershey, PA 17033 USA; 2 Department of Cardiology, Affiliated Hospital of Jiangsu University, Jiefang road 438, Zhenjiang, 212013 Jiangsu Province China

**Keywords:** CD147, Spleen, Inflammation, Cytokines, Monocytes/macrophages, Cerebral ischemia

## Abstract

**Background:**

The splenic inflammatory response after cerebral ischemia has been implicated in secondary brain injury. We have recently reported that CD147 plays an important role in driving brain inflammation after ischemic stroke. In this study, we hypothesized that CD147 may play a role in the splenic inflammatory response after cerebral ischemia.

**Methods:**

Transient (60 min) middle cerebral artery occlusion was induced in wild-type mice treated with an anti-CD147 antibody (αCD147) 1 h before ischemia onset. The splenic inflammatory response was evaluated at 4 and 24 h, representing the peak and early stage of splenic inflammatory activation in this model. Changes in mRNA and protein expression of CD147 and inflammatory markers were measured using RT-qPCR and western blot, respectively. Immune cells in the spleen and brain were measured using flow cytometry.

**Results:**

CD147 expression was rapidly upregulated in the spleen at 4 and 24 h after ischemia onset. The splenic inflammatory response induced by cerebral ischemia was inhibited by αCD147 treatment as demonstrated by the reduced expression of cytokines (TNFα, IL-6, IL-1β) and monocyte chemoattractant protein-1 (MCP-1) in the spleen at 4 and 24 h after ischemia onset. Furthermore, reduced expression of Ly-6C and CCR2 coincided with a decrease in the number of Ly-6C^high^ MMs subset in the spleen at 4 h after ischemia onset. This suggests αCD147 treatment abrogates cerebral ischemia-induced inflammatory activation of splenic monocytes/macrophages (MMs). In addition, the experiment in splenectomized mice showed the spleen as the major source of infiltrated Ly-6C^high^ MMs subset in the ischemic brain and that brain infiltration of Ly-6C^high^ MMs was reduced by αCD147 treatment. These results reveal CD147 as a key mediator of the spleen’s inflammatory activation in response to cerebral ischemia.

## Introduction

Cerebral ischemia not only produces local brain damage, but also has a profound impact on the peripheral immune system. The splenic inflammatory response is rapidly activated following cerebral ischemia and is implicated as a major contributor to secondary ischemic brain damage [1–5]. Previous studies have shown that surgical removal of the spleen 2 weeks prior to ischemia onset reduced ischemic brain injury in both permanent and transient cerebral ischemia models and reduced the infiltration of immune cells into the ischemic brain including a decrease in infiltrated leukocytes and reactive microglia and a reduction of proinflammatory cytokines [6–8]. In addition, splenic irradiation after ischemia onset suppressed the release of splenic immune cells and decreased immune cell brain infiltration and tissue infarction [9]. However, the molecular mechanisms underlying splenic inflammatory activation in response to cerebral ischemia remain poorly understood. A better understanding of the molecular mechanisms involved would aid in developing novel therapies for ischemic brain injury [10].

CD147 (cluster of differentiation 147), a cell surface glycoprotein, has recently been shown to be an important mediator of inflammatory and immune responses [11]. CD147 is expressed in many cell types and widely distributed in various organs including the brain, liver, spleen, intestine, and kidney [11]. Therapeutic targeting of CD147 has been shown to reduce inflammation and disease severity in experimental models of human diseases such as rheumatoid arthritis, asthmatic lung inflammation, myocardial ischemia/reperfusion injury, multiple sclerosis, and experimental autoimmune encephalomyelitis [12–16]. Our recent study has shown that pharmacological inhibition of CD147 ameliorates acute ischemic brain injury with a decrease in infiltrating immune cells including neutrophils, T cells, macrophages, and activated microglia in the mouse brain after focal cerebral ischemia [17]. In this study, we aimed to further investigate the role of CD147 in the splenic inflammatory activation in response to focal cerebral ischemia.

## Materials and methods

### Animals and ethics statement

Male C57BL/6 mice (8–10 weeks, Jackson Laboratories, Bar Harbor, ME) were used in this study. Animal experiments were performed in accordance with the National Institutes of Health Guide for the Care and Use of Laboratory Animals, and the animal protocols were approved by the Institutional Animal Care and Use Committee at Penn State University College of Medicine. Results are reported in accordance with the ARRIVE guidelines [18]. The number of animals used in each group and the total number of animals used in this study are summarized in Additional file 1: Table S1.

### Focal cerebral ischemia model and antibody treatment

Focal cerebral ischemia was induced in mice using a 1-h transient middle cerebral artery occlusion (tMCAO) as we described previously [19]. Sham-operated animals underwent anesthesia and exposure of the arteries but without the suture insertion into the MCA. Rectal temperature was maintained at 37.0 ± 0.5 °C throughout the surgical procedure with a feedback-regulated heating pad. Regional cerebral blood flow (CBF) was monitored in all animals before cerebral ischemia onset, 5 min after ischemia onset and 15 min after reperfusion using laser Doppler flowmetry (MSP300XP; AD Instruments Inc). A CBF reduction of > 85% during tMCAO and a CBF recovery of > 80% after reperfusion were confirmed in all stroke animals. After surgery, the mice were randomly assigned to the following treatment groups: a rat anti-mouse CD147 monoclonal antibody (RL73.2, eBbioscience, named αCD147 mAb throughout this article) or isotype control antibody (rat IgG2a) administered via tail vein injection in 100-μL volume of PBS. This anti-CD147 antibody has been well characterized to block CD147 function in various mouse models [12–17]. In the 4 and 24 h experiments, a single dose of antibody was given 1 h before ischemia onset. In the 72-h experiments, antibody treatment was initiated at 1 h and repeated at 24 and 48 h after ischemia onset.

### Splenectomy

Splenectomy (Spx) was performed according to an established protocol [20]. Briefly, under anesthesia a left flank skin incision (~ 2.5 cm long) was made and the spleen was exposed and pulled onto the exterior surface of the peritoneum with blunt forceps. The splenic blood vessels were tied off with sutures and the spleen was removed. The abdominal wall and the skin incision were closed with sutures. Sham operations were also performed where the spleen was exteriorized and then reinserted into the abdominal cavity. Buprenorphine (0.1 mg/kg, s.c.) was given immediately after surgery and then every 8 h for 3 days. Mice were allowed to recover for 14 days before undergoing cerebral ischemia.

### Isolation of immune cells from mouse spleen and brain

Spleen and brain immune cells were isolated as we described previously [8, 17]. After transcardial perfusion with normal saline (30 ml/mouse), the spleen and brain were harvested for cell isolation. Whole splenocytes were prepared by gentle grinding of spleens between frosted glass slides and then by forcing the tissues through a fine wire mesh. The erythrocytes were lysed using 1× RBC Lysis Buffer (BioLegend) and single-cell suspensions were collected by passing the tissue through a 100-μm nylon mesh. Collected splenocytes were washed twice in PBS, counted, and resuspended in 2 ml PBS containing 10% FBS at a concentration of 10^7^ cells/ml. Brains were separated into two hemispheres (without the brainstem and cerebellum) and cut into small pieces (1–2 mm^3^) and then digested in RPMI-1640 containing type IV collagenase (1 mg/ml, Sigma-Aldrich) and DNase I (50 μg/ml, Roche Diagnostics) at 37 °C for 45 min in a shaker. Dissociated samples were pressed through a 100-μm cell strainer (Falcon). The obtained immune cells were separated by 37–70% Percoll (GE Healthcare) density gradient centrifugation. The cells were removed from the interface and washed twice with PBS, counted, and resuspended in FACS buffer (2% FBS, 0.02% sodium azide in PBS).

### Flow cytometric analysis

The distribution of immune cells including Ly-6C+ monocyte/macrophage (MM) subsets in the spleen and brain were analyzed by flow cytometry according to a published method [21, 22]. The isolated cells from the spleen and brain were suspended in FACS buffer (2%FBS, 0.02% sodium azide in PBS) at 10^8^ cells/ml and incubated with rat anti-mouse CD16/32 mAb (Mouse BD Fc Block™) for 20 min on ice, and immune cells were enriched with anti-mouse CD45 microbeads (Miltenyi Biotec Inc) according to the manufacturer’s instructions. Then, single-cell suspensions (100 μl/FACS tube) were stained with (i) a cocktail of phycoerythrin-conjugated antibodies (Lin-PE) against T cells (CD90.2, 53-2.1), B cells (B220, RA3-6B2), Pan natural killer cells (CD49b, DX5) and NK1.1 (PK136), and granulocytes (Ly-6G, 1A8); (ii) allophycocyanin-conjugated CD11b (M1/70; CD11b-APC); (iii) phycoerythrin-Cy5 (PE-Cy5)-conjugated CD11c (N418) and F4/80 (BM8); and (iv) fluorescein isothiocyanate-conjugate Ly-6C (HK1.4; Ly6C-FITC). The region (R1) selected with Lin-PE^low^/CD11b-APC^high^ was gated as monocytes/macrophages (MMs) for further analysis of the distribution of Ly-6C^low^ and Ly-6C^high^ MM subsets (as shown in Fig. 2c). Debris and dead cells were gated out of the analysis using forward versus side light scatter (FSC vs. SSC) and 7-AAD staining. Debris and dead cells often have a lower level of forward scatter and are found at the bottom left corner of the density plot. Cells were stained with 7-aminoactinomycinD (7-AAD, a viability dye) in order to discern between living and dead cells*.* Flow cytometry was performed on a Becton Dickinson FACS Calibur, and data was analyzed with CellQuest Pro software.

### Fluorescence-activated cell sorting

Fluorescence-activated cell sorting (FACS) of live cells was performed by BD FACSAria II cell sorter using FACSDiva software (BD Biosciences). Isolated mouse splenocytes and brain immune cells were suspended in the PBS containing 2% FBS at a concentration of 1 × 10^7^ cells/ml. Dead cells and debris were gated out of the analysis as described above. A population of splenic monocytes/macrophages (both CD11b^high^/Ly-6C^high^ and CD11b^high^/Ly-6C^low^ subsets) were sorted based on fluorescent labeling and gating (as shown in Fig. 2c). A population of brain microglia (CD11b + CD45^low^) and macrophages (CD11b + CD45^high^) were sorted based on gating strategy as described in Additional file 1: Figure S1.

### Real-time quantitative RT-PCR (RT-qPCR)

Total RNA was extracted from isolated cells (splenocytes, sorted cells) and cerebral cortices (bregma + 2 to − 3 mm) using Tri reagent (MRC, OH). cDNA was synthetized with iScript reverse transcription supermix for qRT-PCR (Bio-Rad). qRT-PCR was conducted with cDNA in duplicate reactions using the Maxima SYBR Green/ROX qPCR Master Mix (2×) (Thermo Scientific). The reactions were performed in 20-μl total volume and incubated at 50 °C for 2 min and then at 95 °C for 10 min followed by 40 cycles of 15 s at 95 °C and 1 min at 60 °C. All samples were run in triplicate. The relative mRNA level of each gene was normalized to that of the housekeeping gene GAPDH. The sequences of the primers used are shown in Table 1.

**Table 1:** Sequences of PCR primers used in this study

Gene	Forward	Reverse
CD147	5′-GACACTGGGGAAGAAGAGGC-3′	5′-GCAGTGAGATGGTTTCCCGA-3′
TNF-α	5′-AGTCCGGGCAGGTCTACTTT-3′	5′-ACCCTGAGCCATAATCCCCT-3′
IL-1β	5′-ATGCCACCTTTTGACAGTGATG-3′	5′-GCAGCCCTTCATCTTTTGGG-3′
IL-6	5′-CCTTCCAGGATGAGGACATGA-3′	5′-TGAGTCACAGAGGATGGGCTC-3′
Arg1	5′-TCACCTGAGCTTTGATGTCG-3′	5′- CTGAAAGGAGCCCTGTCTTG-3′
MCP-1	5′-CCCCAAGAAGGAATGGGTCC-3′	5′-GTGCTGAAGACCTTAGGGCA-3′
Ly6C	5′-ACTGTGCCTGCAACCTTGT-3′	5′-GCTGGGCAGGAAGTCTCAAT-3′
CCR2	5′-ATCCACGGCATACTATCAACATC-3′	5′-CAAGGCTCACCATCATCGTAG-3′
iNOS	5′-CAAGCACCTTGGAAGAGGAG-3′	5′- AAGGCCAAACACAGCATACC-3′
GAPDH	5′-GCGAGATCCCGCTAACATCA-3′	5′-CTCGTGGTTCACACCCATCA-3′

### Western blot analysis

Proteins were extracted from isolated splenocytes and cerebral cortices (bregma + 2 to − 3 mm) as we described previously [8, 17]. Isolated splenocytes were lysed in RIPA buffer (50 mM Tris-HCl pH 7.2, 150 mM NaCl, 1% NP40, 0.1% SDS, 0.5% DOC, 1 mM PMSF, 25 mM MgCl2) with freshly added protease inhibitor cocktail (Sigma-Aldrich). The cerebral cortices of each hemisphere (bregma + 2 to − 3 mm) were homogenized in RIPA buffer with freshly added protease inhibitor cocktail and centrifuged at 10,000 rpm for 10 min at 4 °C. The supernatants were used for western blot analysis. The primary antibodies used are as follows: CD147 (1:1000; Ab188190, Abcam), CCR2 (1:1000, ab32144, Abcam), Ly-6C (1:800, sc-271811, Santa Cruz), and β-actin (1:4000; A2066, Sigma). Samples were developed by electrophoresis on 4–15% polyacrylamide gel (BioRad). Immunopositive bands of horseradish peroxidase-conjugated secondary antibodies were detected with an ECL system (GE Healthcare) and exposure to ECL Hyperfilm. Semi-quantitation of immunoblots was analyzed by densitometry.

### Statistical analysis

GraphPad Prism 5 software package was used for statistical analysis. Data from individual experiments are presented as mean and standard deviation (means ± SD). Unless otherwise indicated, multiple comparisons were made using a one-way analysis of variance (ANOVA) followed by the Bonferroni post hoc test. If only two groups were compared, unpaired, two-tailed Student *t* test was applied. *p* < 0.05 was considered statistically significant.

## Results

### Inhibition of CD147 reduces the early splenic inflammatory response after tMCAO

**Fig. 1:**
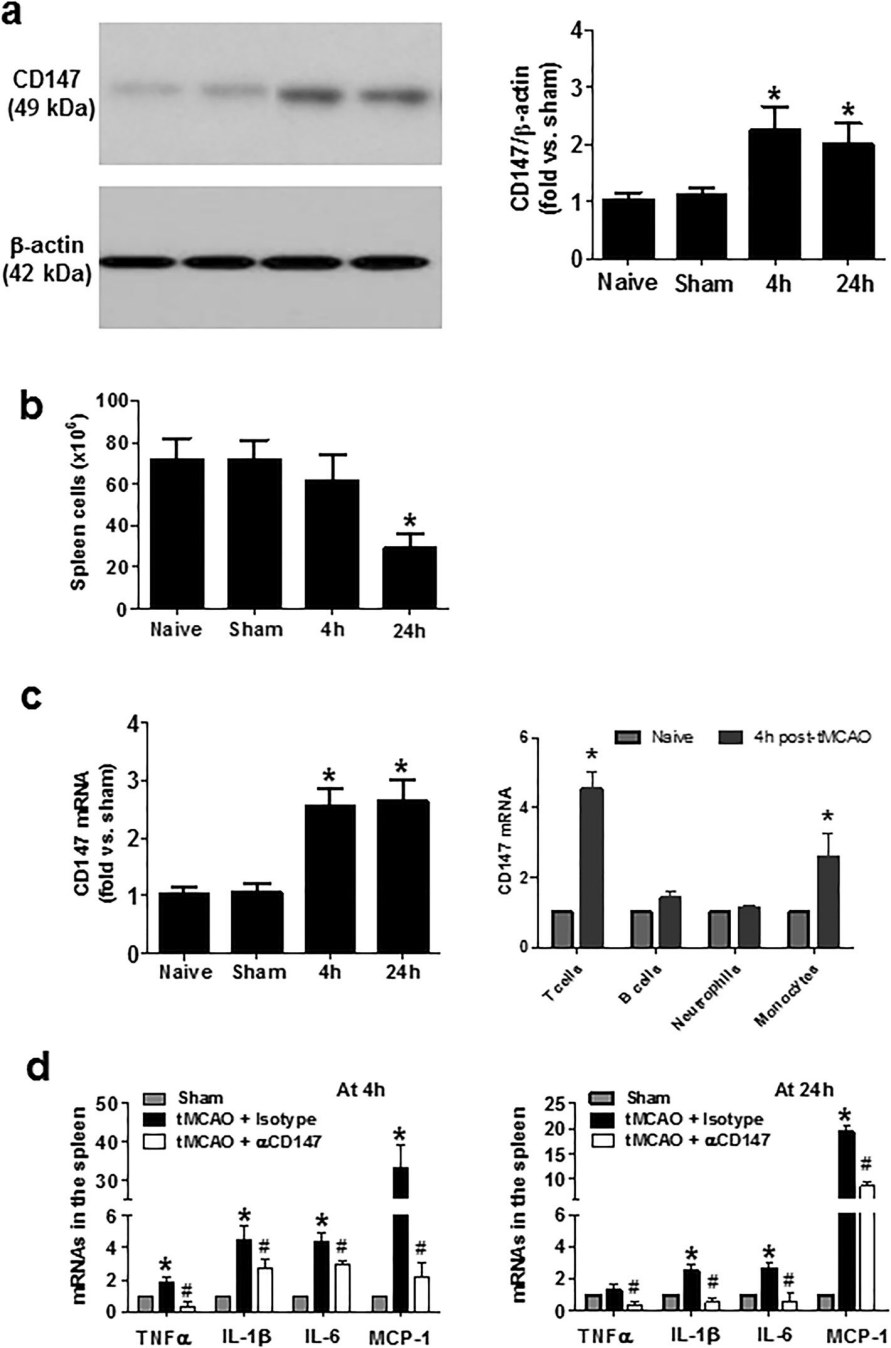
Focal cerebral ischemia and reperfusion induces CD147 expression and early inflammatory response in the spleen. **a** Representative western blot images (left) and semi-quantitation (right) showing CD147 protein levels detected in isolated splenocytes from the following groups (*n* = 5 mice/group): naïve (no surgery), sham surgery, and tMCAO at 4 or 24 h tMCAO. **b** The number of isolated splenocytes from each of the above indicated groups was counted using a hemocytometer. **c** RT-qPCR analysis of CD147 mRNA levels detected in isolated whole splenocytes at 4 or 24 h after tMCAO, and in the different subpopulations of splenocytes (T or B cells neutrophils, monocytes) sorted by FACS at 4 h after tMCAO. *p < 0.05 vs. naïve or sham controls. **d** RT-qPCR analysis of mRNA levels of proinflammatory genes (TNF-α, IL1β, IL6, MCP-1) detected in isolated splenocytes from the following groups (*n* = 5 me/group): sham surgery, tMCAO + isotype, and tMCAO + αCD147. *p < 0.05 vs. sham; ^*#*^*p* < 0.05 vs. tMCAO + isotype

We have previously shown that CD147 expression rapidly increased in the post-ischemic brain after cerebral ischemia [19], but whether it alters CD147 expression in the spleen remains unknown. Using whole splenocytes isolated from the different groups of mice studied, western blot analysis showed that CD147 protein was detectable at low levels in the splenocytes from mice with no surgery (naïve) and sham operation, but significantly increased at 4 and 24 h after ischemia onset (Fig. 1a). Of note, the number of spleen cells was mildly reduced at 4 h but markedly reduced at 24 h after cerebral ischemia (Fig. 1b). RT-qPCR showed that CD147 mRNA expression significantly increased in the isolated whole splenocytes at 4 and 24 h after ischemia onset (Fig. 1c). By RT-qPCR analysis of the FACS-sorted spleen cells, we further found that CD147 mRNA expression was differentially increased in the different subpopulations of splenocytes, with a marked increase in T cells and monocytes and a mild increase in B cells at 4 h after ischemia onset. Unexpectedly, the CD147 mRNA increase was not observed in the sorted neutrophils, possibly due to neutrophil oxidative burst and apoptosis caused by the cell sorting procedure. RT-qPCR showed that the expression levels of proinflammatory cytokines (IFN-γ, TNF-α, IL-1β) and the chemokine (MCP-1) mRNAs were significantly increased at 4 and 24 h after ischemia, and their increases were profoundly inhibited by CD147 inhibition (Fig. 1d).

### Inhibition of CD147 reduces the early proinflammatory activation of splenic monocytes/macrophages after tMCAO

**Fig. 2:**
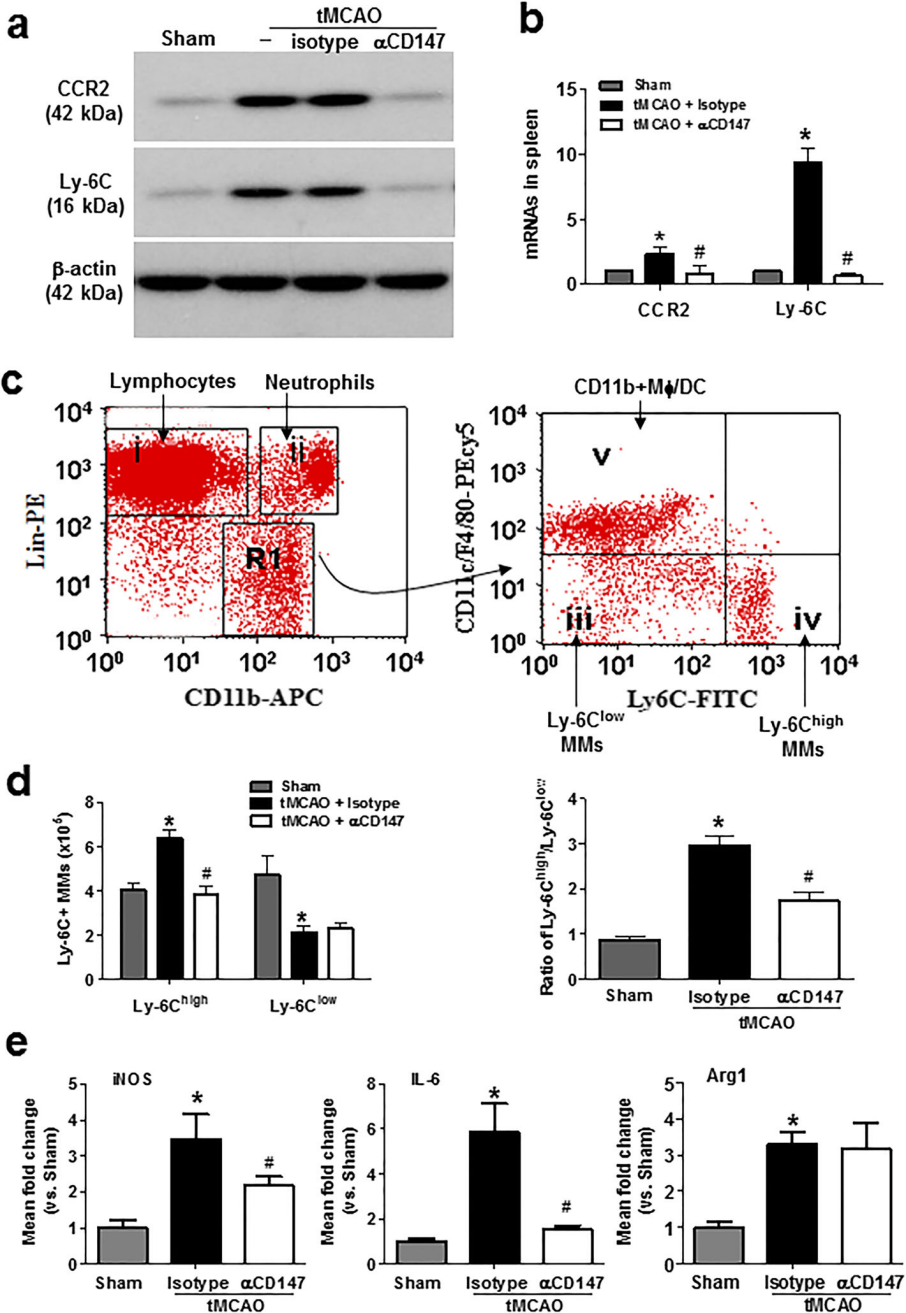
Inhibition of CD147 attenuates early proinflammatory activation of Ly-6C+ monocytes/macrophages (MMs) in the spleen at 4 h tMCAO. **a** Representative western blot images showing protein levels of CCR2 and Ly-6C detected in isolated splenocytes from the following groups (*n* = 5 mice/group): sham, tMCAO, tMCAO + isotype, and tMCAO + αCD147. Similar results were obtained in three independent experiments. No difference was noted between tMCAO and +iso groups. **b** RT-qPCR analysis of mRNA levels of CCR2 and Ly-6C detected in isolated splenocytes from the following groups (*n* = 5 mice/group): sham surgery, tMCAO + isotype, and tMCAO+αCD147. *p < 0.05 vs. sham control; ^*#*^*p* < 0.05 vs. tMCAO + isotype. **c** Representative dot plots illustrating the flow cytometry gating strategy for identification of immune cell subsets in the spleen. Monocyte subsets in the spleen were identified by selecting the gate (R1) APC-CD11b^high^ and PE-Lin^low^ and further separated by CD11c/F4/80-PeCy5 and Ly-6C-FITC. **d** Flow cytometric quantification of Ly-6C^high^ and Ly-6C^low^ monocyte subsets. The number and ratio of Ly-6C^high^ and Ly-6C^low^ monocyte subsets in spleen were measured in the following groups (*n* = 5 mice/group): sham surgery, tMCAO + isotype, and tMCAO + αCD147. *p < 0.05 vs. sham; ^*#*^*p* < 0.05 vs. tMCAO + isotype. **e** RT-qPCR analysis of mRNA levels of iNOS, IL-6, and Arg1 detected in the FACS-sorted splenic monocytes from the indicated groups of mice (*n* = 6/groups). *p < 0.05 vs. sham control; ^*#*^*p* < 0.05 vs. tMCAO + isotype

There are two main monocyte subsets in humans and mice based on their expression of Ly6C and CCR2: proinflammatory Ly-6C^high^ CCR2^high^ and anti-inflammatory Ly-6C^low^ CCR2^low^ subsets [22, 23]. Western blot analysis showed that the protein expression levels of both Ly-6C and CCR2 in isolated whole splenocytes were rapidly increased at 4 h after ischemia onset, and their increases were almost completely suppressed by inhibition of CD147 (Fig. 2a). Similarly, quantitative PCR showed that mRNA expression of both Ly-6C and CCR2 in isolated whole splenocytes rapidly increased at 4 h after ischemia onset and was attenuated by inhibition of CD147 (Fig. 2b). Furthermore, flow cytometric analysis of isolated whole splenocytes was performed to assess the distribution and expression of Ly-6C+ monocyte subsets in the spleen (Fig. 2c). The proportion of the proinflammatory (M1-type: CD11b+/Ly-6C^high^) monocyte subset in the spleen rapidly increased at 4 h after ischemia onset. This increase was blunted by αCD147 treatment (Fig. 2d). However, the anti-inflammatory (M2-type: CD11b+/Ly-6C^low^) monocyte subset was not significantly altered by either cerebral ischemia or αCD147 treatment at this time point. Furthermore, we assessed activation state of splenic monocytes after tMCAO. Splenic monocytes (including both Ly-6C^high^ and Ly-6C^low^ subsets) were sorted for qRT-PCR analysis. The data showed that mRNA levels of the M1-type monocyte markers such as inducible nitric oxide synthase (iNOS) and interleukin 6 (IL-6) and the M2-type marker Arginase 1 (Arg1) were significantly increased at 4 h after tMCAO compared with sham controls, and αCD147 treatment significantly reduced iNOS and IL-6 but did not affect Arg1 mRNA levels (Fig. 2e).

### Inhibition of CD147 reduces Ly-6C^high^ monocytes/macrophages in the brain after tMCAO

**Fig. 3:**
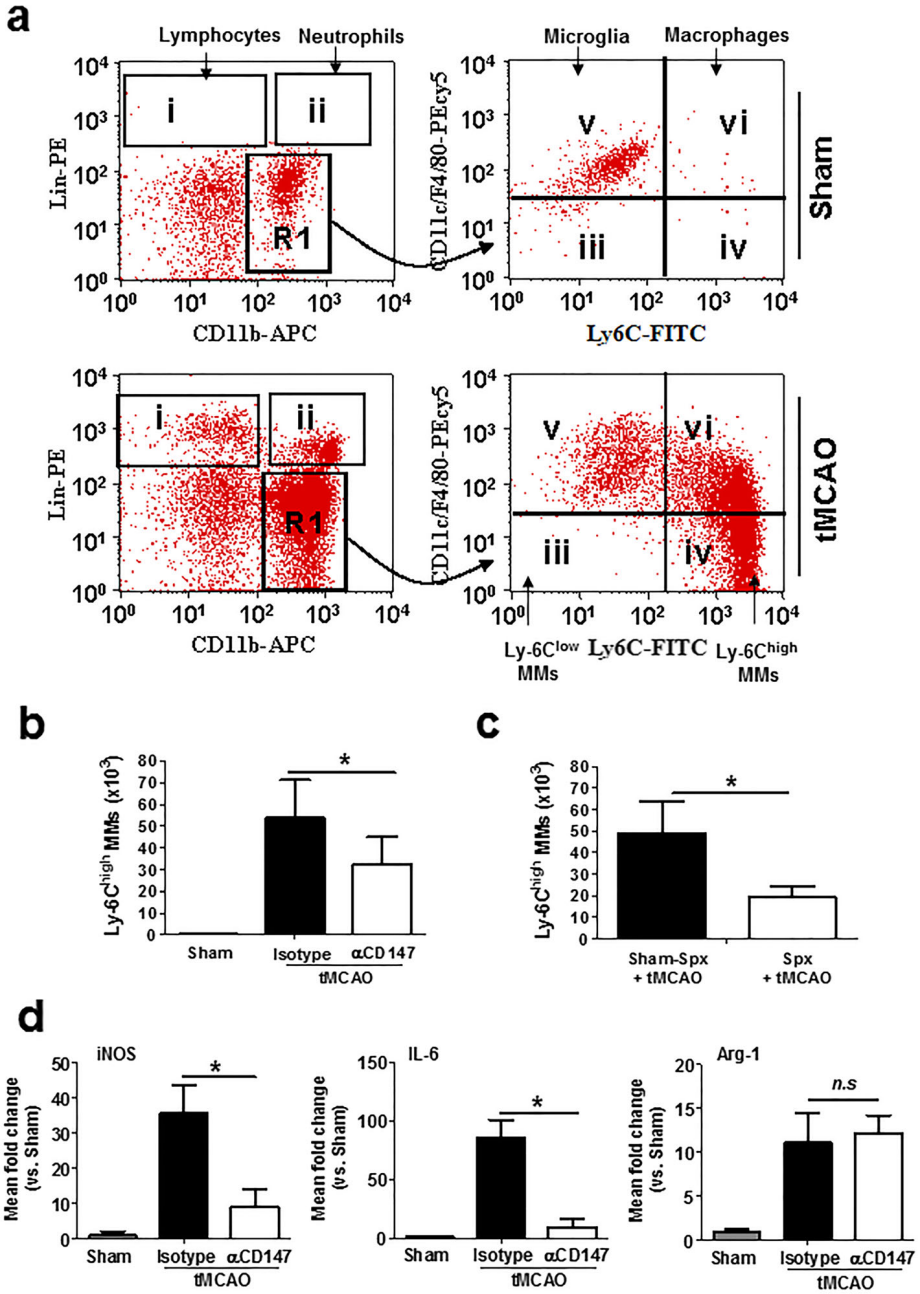
Inhibition of CD147 decreases the deployment and proinflammatory activation of Ly-6C+ monocytes/macrophages (MMs) in the post-ischemic brain 72 h after tMCAO. **a** Representative dot plots illustrate the flow cytometry gating strategy for identification of immune cell subsets in the brain. **b** Flow cytometric quantification of Ly-6C^high^ MMs in the ischemic and non-ischemic (sham) hemispheres from the indicated groups of mice (*n* = 5 mice/group). **c** Flow cytometric quantification of Ly-6C^high^ MMs in the ischemic and non-ischemic (sham) hemispheres from the following groups of mice (*n* = 6/group): Spx + tMCAO, sham-Spx + tMCAO. *p < 0.05. Splenectomy (Spx) and sham-Spx surgery were performed 2 weeks prior to tMCAO. **d** RT-qPCR analysis of mRNA levels of M1 markers (iNOS, IL-6) and M2 marker (Arg1) detected in the FACS sorted brain microglia/macrophages from the indicated groups of mice (*n* = 6/groups). *P < 0.05

Flow cytometric analysis of isolated brain cells was performed to assess the distribution and expression of Ly-6C+ monocytes/macrophage (MMs) subsets in the brain (Fig. 3a). The results showed that (1) both Ly-6C^low^ and Ly-6C^high^ monocyte subsets were absent in the normal non-ischemic brains of sham-operated mice; (2) Ly-6C^high^ cells, including proinflammatory monocytes (Ly-6C^high^/F4/80^dim^) and macrophages (Ly-6C^high^/F4/80^bright^), are markedly increased in the ischemic hemisphere at 72 h after cerebral ischemia while Ly-6C^low^ cells were not altered; and (3) the resident microglia population was more prominent at 72 h after cerebral ischemia than in sham-operated mice. Inhibition of CD147 reduces the Ly-6C^high^ monocytes/macrophages in the brain at 72 h after cerebral ischemia (Fig. 3b). Moreover, we showed that splenectomy (Spx) 2 weeks prior to cerebral ischemia resulted in about 65% reduction of Ly-6C^high^ MMs in ischemic brains compared to sham-Spx ischemic mice at 72 h after cerebral ischemia (Fig. 3c). This indicates that the spleen is the major source of infiltrating monocytes/macrophage (MMs) subsets in the ischemic brain in the focal cerebral ischemia model.

During cerebral ischemia, monocytes accumulate in the ischemic brain and mature to macrophages and microglia-like cells [24]. Furthermore, we assessed activation state of brain microglia/macrophages after tMCAO. Microglia (CD11b + CD45^low^) and macrophages (CD11b + CD45^high^) were sorted for qRT-PCR analysis. The data showed that mRNA levels of the M1 phenotypic markers (iNOS, IL-6) and the M2-type marker (Arg1) were significantly increased at 72 h after tMCAO compared with sham controls, and αCD147 treatment significantly reduced iNOS and IL-6 but did not affect Arg1 mRNA levels (Fig. 3d).

## Discussion

The present study, for the first time, demonstrates that CD147 acts as a key regulator of the splenic inflammatory response during cerebral ischemia. Experimental studies have demonstrated that the spleen displays a biphasic immune response to cerebral ischemia: rapid activation of splenic immune cells (within the first few hours after ischemia onset*)* followed by transient splenic atrophy (within the first few days after ischemia*)* with massive release of immune cells from the spleen into the circulation and subsequent infiltration into the ischemic brain [10, 25]. Activated immune cells in the spleen may also contribute to elevated blood levels of inflammatory cytokines and chemokines during the acute phase of cerebral ischemia [25]. It has been reported that at both 6 and 22 h after cerebral ischemia, activated splenocytes from ischemia-injured mice produce significantly higher levels of tumor necrosis factor-*α* (TNF-*α*), interferon-gamma (IFNγ), interleukin-6 (IL-6), and monocyte chemoattractant protein-1 (MCP-1) compared to splenocytes from non-ischemic mice [1]. The present study demonstrates the following: (1) CD147 expression rapidly increased in the spleen after cerebral ischemia and (2) inhibition of CD147 with αCD147 treatment 1 h prior to ischemia onset profoundly reduced cerebral ischemia-induced proinflammatory gene expression of TNF-*α*, IL-1β, IL-6, and MCP-1 in the spleen at 4 and 24 h after cerebral ischemia. It is less likely that the reduction in the splenic inflammatory response is only a consequence of the neuroprotective effect by αCD147 treatment, because in rodent models of cerebral ischemia, the brain tissue damage is not fully developed during the early hours (0–4 h) after onset of ischemia [26]. These findings support an important role for CD147 in mediating the splenic inflammatory response during the early phase of cerebral ischemia.

There are two subsets of monocytes/macrophages (MMs) in mice: Ly-6C^high^ proinflammatory subset and Ly-6C^low^ anti-inflammatory subset [27]. It has been shown that cerebral ischemia differentially regulates splenic Ly-6C^high^ and Ly-6C^low^ MM subsets in mice [28]. The number of total MMs in the spleen was reduced slightly at 3 h but markedly reduced at 1 and 3 days after (30 min) cerebral ischemia. Correspondingly, the number of Ly-6C^high^ MMs in the spleen rapidly and transiently increased (~ 30%) at 3 h followed by a marked reduction (by 70%) at 1 and 3 days after cerebral ischemia. In contrast, immediate and sustained reduction of Ly-6C^low^ MMs was observed from 3 h to 7 days after cerebral ischemia. In agreement with previous observations, we observed that the number of the splenocytes was slightly reduced (~ 15%↓) at 4 h and continued to decrease at 24 h (~ 60%↓) following (60 min) cerebral ischemia. We therefore chose the 4-h time point after onset of ischemia to study the role of CD147 in modulating splenic Ly-6C^high^ and Ly-6C^low^ MM subsets prior to severe loss of splenocytes. We demonstrate that (1) the number of Ly-6C^high^ MMs in the spleen was significantly increased at 4 h after cerebral ischemia, which is correlated with increased expression of the proinflammatory monocyte markers (Ly-6C and CCR2) in isolated splenocytes from ischemic mice, and (2) these ischemia-induced effects were almost completely prevented by αCD147 treatment 1-h prior to ischemia onset. These findings support an important role for CD147 in mediating the phenotypic shift of splenic MMs towards proinflammatory Ly-6C^high^ MMs subset during the acute phase (first few hours) of cerebral ischemia.

The spleen-derived Ly-6C^high^ monocytes have been implicated in the pathogenesis of ischemia/reperfusion injury in other organs such as the heart, lung, and liver [29–31]. However, the exact role of spleen-derived immune cells including monocytes in cerebral ischemia is still a matter of dispute. Wang et al. [32] has elegantly demonstrated that adoptive transfer of immune subsets 24 h before tMCAO including either non-activated or activated CD4^+^ or CD8^+^ T cells or activated monocytes to splenectomized male or female mice does not exacerbate infarct volume and neurological deficits 4 days after cerebral ischemia. Because many studies have supported the important role of inflammatory cells including T cells and monocytes in cerebral ischemia, the spleen may be required to unleash the deleterious or beneficial effects of adoptively transferred immune cells. A recent study by Garcia-Bonilla et al. has shown that adoptive transfer of LPS-preconditioned monocytes protects the brain from ischemic injury in naïve mice but not in splenectomized mice, and unlike monocytes, macrophages are not neuroprotective [33]. To explain this finding, the authors proposed possible mechanisms: (1) monocytes may need to home to the spleen to become licensed to exhibit their neuroprotective phenotype, and (2) alternatively, adoptively transferred monocytes might instruct other immune cells in the spleen to change their inflammatory phenotype [34]. Therefore, it is possible that splenic MMs, depending on their activation state, instruct other splenic immune cells poised to participate in the inflammatory response after cerebral ischemia to exhibit either a proinflammatory or anti-inflammatory phenotype. In the present study, we did not specifically address the deleterious or beneficial effects of splenic monocytes in cerebral ischemia, but our findings reveal that (1) splenic monocytes are the major source of proinflammatory Ly-6C^high^ MMs deployed to the ischemic brain, and (2) inhibition of CD147 significantly decreased the deployment and proinflammatory activation of spleen-derived Ly-6C^high^ MMs to the ischemic brain after cerebral ischemia. Spleen-derived monocytes could differentiate into macrophages and microglia-like cells in the ischemic brain. Recent studies suggest that microglia and monocytes/macrophages in the ischemic brain can polarize to the classic proinflammatory type (M1-like) or alternative protective type (M2-like), thereby exerting a detrimental or protective effect in cerebral ischemia [33]. Using RT-qPCR analysis of the FACS sorted cells, we demonstrate that inhibition of CD147 significantly decreased tMCAO-induced expression of M1-type (iNOS, IL-6) markers in both the splenic Ly-6C+ monocytes (at 4 h) and the brain microglia/macrophages (at 72 h) but did not alter tMCAO-induced M2 marker (Arg1). These findings suggest that attenuated neuroinflammation by inhibition of CD147 could be associated with a reduced M1-like/M2-like ratio of splenic monocytes and brain microglia/macrophages during cerebral ischemia.

## Conclusion

Because splenic inflammatory activation contributes to both enhanced proinflammatory cytokines in the blood and the massive release of spleen-derive immune cells including proinflammatory Ly-6C^high^ MMs into the circulation during the early phase (within first 4 h to 24 h) after cerebral ischemia, it is important to understand the molecular mechanisms and to therapeutically manipulate the early splenic inflammatory activation. The present study reveals for the first time that CD147 acts as a key mediator of the early splenic inflammatory activation in response to cerebral ischemia. Our present and previous studies demonstrate that inhibition of CD147 simultaneously attenuates splenic inflammation and neuroinflammation; therefore, CD147 might represent a promising therapeutic target to ameliorate peripheral and central inflammation and subsequent ischemic brain injury.

## Additional file

**Additional file 1: Figure S1.** Representative dot-plots illustrate the flow cytometry gating strategy for identification of microglia and macrophages in the brain. Ly-6G- immune cells were selected for FACS analysis and both microglia (CD11b + CD45low) and macrophages (CD11b + CD45high) were sorted. All cells were sorted twice to maximize cell purity and used for RT-qPCR. **Table S1.** The number of animals used in this study.

## Data Availability

All relevant data are included in this manuscript. Supplemental Material is available online.

## Abbreviations

CCR2: C-C chemokine receptor type 2
CD147: Cluster of differentiation 147
IL-1β: Interleukin 1 beta
IL-6: Interleukin 6
iNOS: Inducible nitric oxide synthase
Ly-6C: Lymphocyte antigen 6 complex
MCP-1: Monocyte chemoattractant protein-1
TNFα: Tumor necrosis factor alpha
