# Supplementary material for: CD147 as a key mediator of the spleen inflammatory response in mice after focal cerebral ischemia
Source: J Neuroinflammation. 2019 Oct 30;16:198. doi: 10.1186/s12974-019-1609-y (PMC6822438; doi:10.1186/s12974-019-1609-y)
Supplement: Supplementary file 1 — Additional file 1: Figure S1. Representative dot-plots illustrate the flow cytometry gating strategy for identification of microglia and macrophages in the brain. Ly-6G- immune cells were selected for FACS analysis and both microglia (CD11b + CD45low) and macrophages (CD11b + CD45high) were sorted. All cells were sorted twice to maximize cell purity and used for RT-qPCR. Table S1. The number of animals used in this study. [file 12974_2019_1609_MOESM1_ESM.docx]

**Additional file**

**Additional file 1:
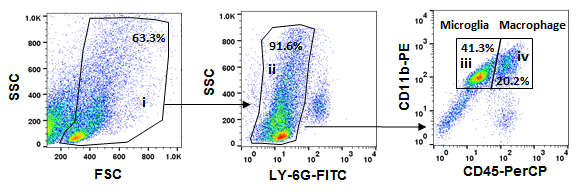
Figure S1.** Representative dot-plots illustrate the flow cytometry gating strategy for identification of microglia and macrophages in the brain. Ly-6G- immune cells were selected for FACS analysis and both microglia (CD11b+CD45low) and macrophages (CD11b+CD45high) were sorted. All cells were sorted twice to maximize cell purity and used for RT-qPCR.

**Additional file 1: Table S1.** The number of animals used in this study

Experiments Groups No./group Time point Total No.

Fig.1a, 1b, 1c Naïve, Sham, 5 4h or 24h 20

(WB, RT-PCR) MCAO (4h),MCAO (24h)

Fig.1d Sham, MCAO + Iso

(RT-PCR) MCAO + αCD147 5 4h, 24h 30

Fig. 2a, 2b Sham, MCAO, MCAO + Iso, 5 4h 20

(WB, RT-PCR) MCAO + αCD147

Fig. 2c, 2d Sham, MCAO + Iso 5 4h 15

(FCM) MCAO + αCD147

Fig. 2e Sham, MCAO + Iso 6 4h 18

(RT-PCR) MCAO + αCD147

Fig. 3a, 3b Sham, MCAO + Iso 5 72h 15

(FCM) MCAO + αCD147

Fig. 3c. Sham-Spx + MCAO 6 72h 12

(FCM) Spx + MCAO

Fig. 3d Sham, MCAO + Iso 6 72h 18

(RT-PCR) MCAO + αCD147

**Total 148**
